# SELEX tool: a novel and convenient gel-based diffusion method for monitoring of aptamer-target binding

**DOI:** 10.1186/s13036-019-0223-y

**Published:** 2020-01-13

**Authors:** Qingxiu Liu, Wei Zhang, Siying Chen, Zhenjing Zhuang, Yi Zhang, Lingli Jiang, Jun Sheng LIN

**Affiliations:** School of Medicine, Huaqiao University, 269 Chenghua Rd, Fengze, Quanzhou, 362021 Fujian China

**Keywords:** Aptasensor, Monitor, Aptamer, Interaction, Visualization, Diffusion

## Abstract

**Background:**

Aptamers, single-stranded DNAs or RNAs, can be selected from a library containing random sequences using a method called Systematic Evolution of Ligands by EXponential Enrichment (SELEX). In SELEX, monitoring the enriching statuses of aptamer candidates during the process is a key step until today. Conformational change of an aptamer caused by target-binding in gel can be used to indicate its statuses of binding.

**Results:**

In this study, an easy-to-implement gel-based diffusion method (GBDM) was developed to monitor the interaction between enriched aptamer candidates and their targets. In order to prove the concept, characterization of aptamers targeting their targets including protein (thrombin) and non-protein molecules (acetamiprid, ATP, atrazine, profenofos and roxithromycin), respectively, were performed using mini gels. Our method has advantages over the common methods including easy performed with labor- and time- saving in experimental operation. The concept has been proven by monitoring enrichment of dynamic aptamer candidate libraries targeting a small molecule 2,2-bis(4-chlorophenyl) acetic acid (DDA) during SELEX process. A mini gel cassette was designed and fabricated by our laboratory to make mini agarose gels for diffusion with different directions.

**Conclusions:**

These results indicate that GBDM, in particular, chasing diffusion is suitable for monitoring the interaction between enriched aptamer candidates and their targets. These pioneering efforts are helpful for novel aptamer selection by breaking through the technical bottleneck of aptamer development and helpful for development of novel aptasensors.

## Background

Systematic Evolution of Ligands by EXponential Enrichment (SELEX) is a common method currently used for isolating high-affinity single-stranded (ss) DNAs or RNAs from a large library with random sequences [1–3]. These SELEX-derived DNAs and RNAs named aptamers, can be selected against a broad range of targets (e.g. proteins, cells, virus, microorganisms, toxins and chemical compounds) [4–6]. Aptamers have high affinity and specificity to their target molecules, similar to antigen-antibody interaction [7], therefore, they are also called “chemical antibodies”. Aptamers offer advantages over antibodies including smaller size, higher pH and thermal stability, lower immunogenicity and toxicity, better tissue penetration, lower synthesis costs and easier conjugation or modification [8]. In addition to their widespread use as stand-alone affinity binding reagents in analytical chemistry, aptamers have been engineered into a variety of ligand-specific biosensors, termed aptasensors [9]. Aptasensors can be designed to integrate with a variety of readout methods, such as electrochemical aptasensors [10–12] fluorescent aptasensors [13, 14], label-free aptasensors [15, 16], and aptasensors designed to interface with nucleic acid signaling cascades [17, 18] or specifically for detection of targets such as pathogens [19, 20] or small molecules [7, 21].

Compared with antibody-based immunosensors and enzyme-based general biosensors, aptasensors exhibited better specificity and sensitivity, higher stability, greater flexibility, easier artificial synthesis as well as longer shelf life [22–24]. Moreover, the production of conventional animal-based specific monoclonal antibodies utilized for immunosensors is tedious, very expensive and challenging, and there are batch-to-batch variations, which further limits their applications [25]. Therefore, benefits from the booming development of various aptasensors for fast, selective, sensitive and on-site targets detection have attracted much attention. Aptamers as the molecular recognitions are at the center of aptasensors, however, up to date, there are only several target-specific aptamers developed for development of aptasensors and some of those aptamers lack selectivity, which is not able to distinguish among analogues [26]. Thus, more highly target analytes-specific aptamers need to be selected.

Since SELEX emerged in 1990, many selection methods have been developed for isolating aptamers such as purified-protein-based SELEX, whole-cell-based SELEX, capillary electrophoresis (CE)-based SELEX, Capture-SELEX, and so on [27–30]. Due to the low successful rates for aptamer selection, several factors in selection process have still been studying [31–33]. Importantly, monitoring enriching progress of candidate aptamers has always been the key and restrictive step in SELEX process. To date, many methods such as dot blotting, enzyme-linked oligonucleotide assay (ELONA), surface plasmon resonance (SPR), electrophoretic mobility shift assay (EMSA) and real-time quantitative PCR (qPCR) have been evaluated for monitoring the enriching situation of library (Table 1). During the partition step of Capture-SELEX process [50], for example, candidate molecules that bind to their target can be eluted and theoretically separated from non-bound ssDNA molecules. In practice, however, eluted DNA sub-library almost always contain more or less non-candidate sequences. This is why iterative enrichment is needed in the SELEX process. Monitoring of overall signal of the eluted DNA sub-library results in confusion with the non-specific eluates. On the other hand, detection of candidate-target binding signal can better monitor the progress of the enrichment. We have investigated how to measure the interaction between enriched aptamer candidates and their target molecules. Herein, we report a simple gel-based approach, which provides an effective way for monitoring the enrichment of dynamic libraries in the process of SELEX.

**Table 1:** Advantages and disadvantages of reported common methods for monitoring the aptamer-target binding in SELEX rounds

Monitoring methods	Suitable targets	Advantages	Limitations	References
Dot blotting	Protein	Focus on the candidate aptamers binding phase, which shows the real enrichment in SELEX; Relative ease of performance	Sequence labeling required; Not suitable for the target molecules with the same electrostatic charge as NC membrane used	[34]
qPCR	Protein & Small molecule	Relative ease of performance	Focus on the elution phase, which could be confused by non-specific eluates; Error of nonspecific amplification;	[35–38]
EMSA	Protein	Focus on the candidate aptamers binding phase, which shows the real enrichment in SELEX	Sequence labeling required; Labor- and time-consuming; Not suitable for small molecule targets	[39]
Gel-shifting	Protein	Focus on the candidate aptamers binding phase, which shows the real enrichment in SELEX; Easy for performance	Detection in none-binding conditions, I.E. electrophoretic buffer solution; Not suitable for small molecule targets	[34, 36]
ELONA	Protein	Focus on the candidate aptamers binding phase, which shows the real enrichment in SELEX; Relative ease of performance	Sequence labeling required; Labor- and time-consuming; Non-specific binding of candidates to the plate, which confuse the enrichment; Not suitable for small molecule targets	[40]
Agarose gel analysis	Protein & Small molecule	Relative ease of performance	Focus on the elution phase, which could be confused by non-specific eluates; Error of nonspecific amplification	[41]
HTS	Protein & Small molecule	Focus on the enrichment of candidate aptamers according to the SELEX rounds	Expensive cost required; Focus on the elution phase, which could confuse by none-specific elution; Time-consuming for sample preparation	[35, 42]
SPR	Protein & Small molecule	Focus on the candidate aptamers binding phase, which shows the real enrichment in SELEX	Expensive sensor chip required; Labor- and time-consuming	[42, 43]
UV quantification	Small molecule	Ease of performance	Focus on the elution phase, which could confuse by none-specific elution	[44]
Fluorescence quantification	Small molecule	Relative ease of performance	Sequence labeled with fluorophore required; Focus on the elution phase, which could confuse by none-specific elution	[45, 46]
Fluorescence binding assay	Small molecule	Focus on the candidate aptamers binding phase, which shows the real enrichment in SELEX; Relative ease of performance	The autofluorescence of target required	[47, 48]
Gel-elution assay	Small molecule	Focus on the candidate aptamers binding phase, which shows the real enrichment in SELEX	Target-coupled column required; Labor- and time-consuming	[49]
GBDM	Protein & Small molecule	Focus on the candidate-target binding phase, which shows the real enrichment in SELEX; Easy for performance without expensive equipment; Suitable for monitoring every selection step during SELEX process	Overnight diffusion required	Optimized in This work

Notes: *qPCR* real-time quantitative PCR, *EMSA* Electrophoretic mobility shift assay, *ELONA* enzyme-linked oligonucleotide assay, *HTS* High throughput sequencing, *SPR* Surface plasmon resonance, *GBDM* gel-based diffusion method

## Results & discussions

A variety of methods for monitoring of SELEX process have been reported. We have evaluated a range of approaches including the methods of EMSA [34], dot blotting, Eastern blotting and target-capture assay [51], quartz crystal microbalance (QCM) analysis [52], qPCR (data not shown), HTS technology (data not shown), and GBDM in our laboratory. We rank the methods. Chasing diffusion, one of GBDM, is the most practically useful to monitor the interaction between enriched aptamer candidates and their targets round by round during SELEX process.

### A mini gel cassette was designed and fabricated for GBDM

**Fig. 1:**
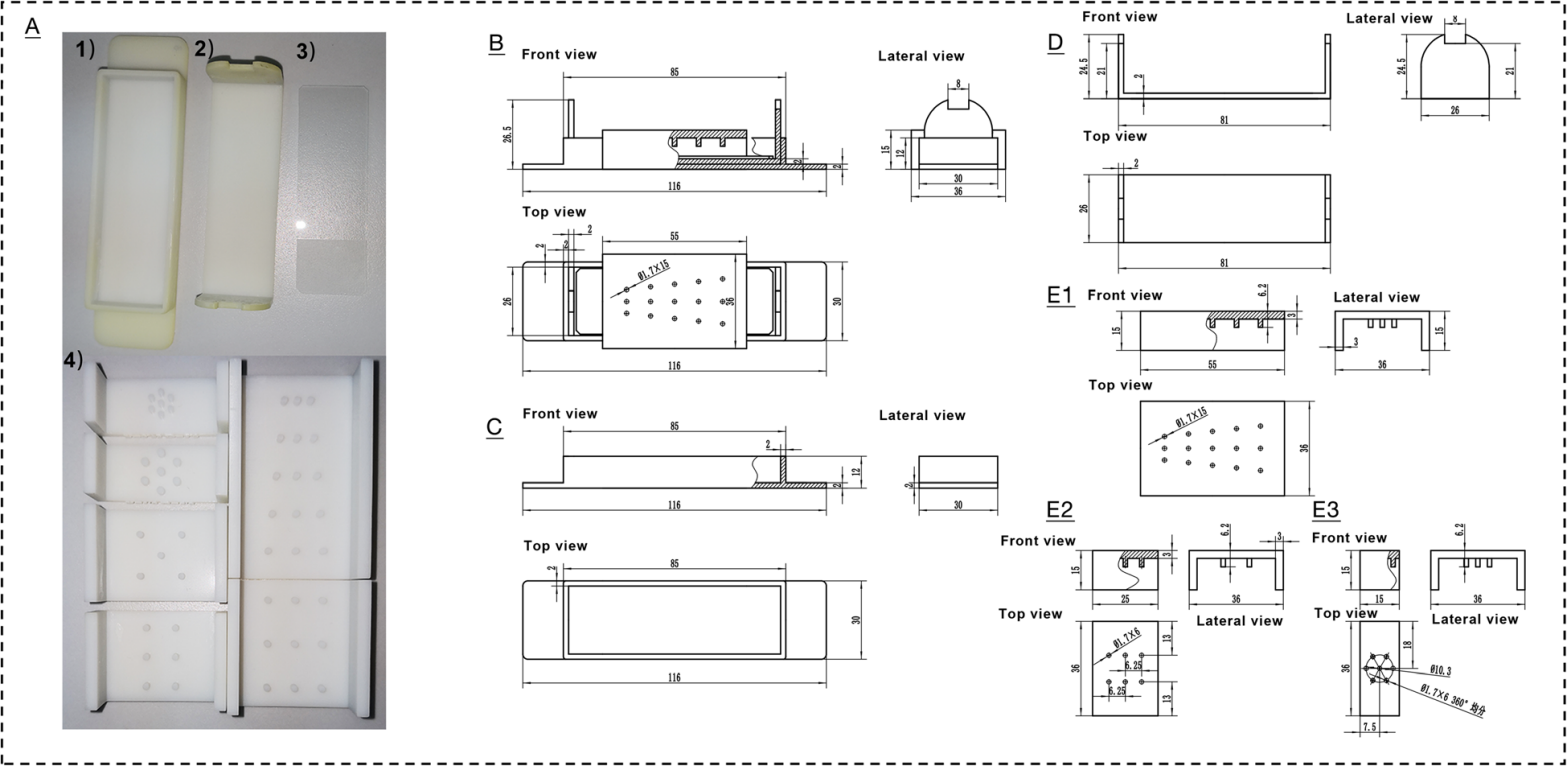
The mini gel cassette and its diagrams. **a** Mini gel cassette with base (**a1**), tray (**a2**), smooth glass plate (**a3**) and a set of hole-making molds (**a4**); **b** Assembly drawing of the invented device. Diagrams of the base (**c**), the tray (**d**) and three representative hole-making molds (**e**). **e1** hole-making mold with well spacing ranging from 3.5 to 7.0; **e2** hole-making mold with six-well (parallel arrangement); **e3** hole-making mold with seven-well (six-well around a centre). Units: mm

A mini gel cassette was designed and fabricated as a tool to cast mini gels used for the purpose of monitoring. The cassette consists of four parts: a base with a concave, a tray, a smooth glass plate (e.g. glass slide commonly used for microscopy), and one piece of a set of hole-making molds (Fig. 1a). In order to ensure a consistent distance between each bottom of the sample wells and the surface of the glass plate, the parts except glass plate were fabricated by 3D printing technology using Stereo Lithography Apparatus. Before making gel, these four parts can be assembled as shown in the diagram in Fig. 1b. The front, top and side views of the base and the tray are shown in Fig. 1c and d, respectively. Hole-making mold is a key aspect determining the diffusion profile of GBDM. The front, top and side views of three representative hole-making molds are shown in Fig. 1e (e1, e2 and e3).

### Excellent performance in double immunodiffusion (DID) assay by using mini gel cassette

DID was commonly used as a screening test for the monitoring of binding of antibody and antigen [53]. For conventional DID assay, the sample wells in gel are typically prepared by punching such as multiple-well patterns after the gel is solidified. These may create a damage on the bottom of wells, which may cause leakage. For our proposed device, a consistent distance between each bottom of the wells and the surface of the glass plate is ensured for preparation the well bottom-flat gels. We make mini gel using the cassette and perform DID assay routinely with mini gel. A representative gel is shown in Additional file 1: Figure S1. Thus, our device is a good alternative tool for immunodiffusion experiments.

### Optimization of conditions for GBDM

Optimization of conditions for GBDM were conducted in four aspects, including well size, well positioning, gel concentration, and diffusion duration.

Giving the fact that 10 μL pipette tips are commonly used in aptamer laboratories. The well size shouldn’t be too large or too small. The optimization results showed that the well sizes in diameter of 0.85 mm (maximum volume of 5.0 μL) and of 1.0 mm (maximum volume of 6.0 μL) were ideal for sample loading (Additional file 2: Table S1). The well spacing is another important aspect for double diffusion. Hole-making molds with different distances between individual wells from 3.5 to 7.0 mm were investigated (Fig. 1e1). The results showed that there was a linear relationship between the intensity of the “precipitation line” and the spacing distance (Additional file 3: Figure S2). Distances of 5.0 and 6.0 mm were optimal for double diffusion.

There was no significant difference in the diffusion situation among different gel concentrations of 2.0% and 3.0% (data not shown) for the purpose of double diffusion. In general, gel concentration of 2.0% is suitable for larger target molecules in molecular weight and 3.0% for smaller target molecules. In addition, there was no significant difference between the diffusion profile of vertically placed gel (needs 5 min incubation in horizontal firstly) and that of horizontal gel (Additional file 4: Figure S3).

### Aptamer-based double diffusion

**Fig. 2:**
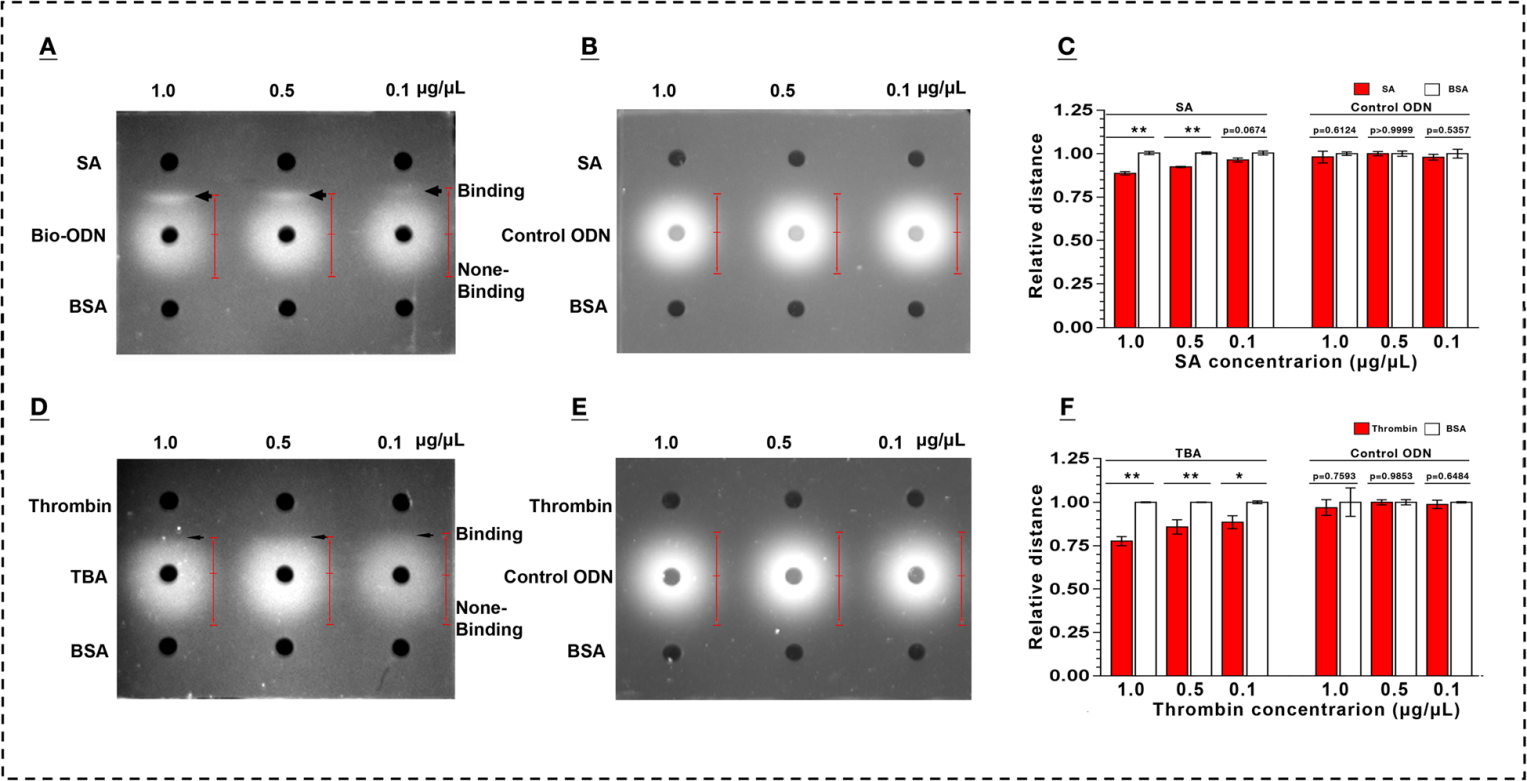
Characterization of oligodeoxynucleotides (ODN)-target binding by double-diffusion in mini-gels. Diffusion profiles of ODN modified with biotin (Bio-ODN) (**a**) and without biotin (control ODN) (**b**) during diffusion toward target streptavidin (SA) and non-target bovine serum albumin (BSA), respectively. Quantitative analysis of diffusion distance of ODN from the central point of different loading wells to their diffusion edge (**c** & **f**). Diffusion profiles of thrombin aptamer (TBA) (**d**) and non-aptamer ODN (control ODN) (**e**) when they diffused toward target thrombin and non-target BSA, respectively. Indication of binding: shortened diffusion distance due to stagnation of diffusion caused by the formation of binding complexes at the interface of the double-diffusion. Arrow heads in **a**: appearance of precipitation line of binding complexes at the interface of the double-diffusion. Thin arrow heads in **d**: interface of the double-diffusion of aptamer and its target; Data represent mean ± SEM. *P < 0.05, **P < 0.01, set control = 1.0. GelRed as the DNA indicator

To test our established system, interactions of thrombin and its binding aptamer (TBA), streptavidin (SA) and biotinylated oligodeoxynucleotides (Bio-ODN) were investigated respectively. TBA/Bio-ODN was stained with GelRed in gel. When the molecules of TBA/Bio-ODN and their targets are loaded into the opposite wells, they diffuse from their respective loading wells. The binding of aptamer-target will halt the each diffusion at their interface, thereby shortening the diffusion distance and even forming a precipitation line. Indeed, image analysis results showed that visible diffusion distance of target-bound aptamer/ODN were significantly shorter than that of target-free aptamer/ODN, even “precipitation lines” could be obviously observed in Bio-ODN and SA binding assay with dose-dependent manner (Fig. 2a & c). This is similar to double-immunodiffusion assay for detection of antibody-antigen interaction. Although none of “precipitation line” was found in thrombin and TBA binding groups, the visible diffusion distance of TBA to thrombin is consistently shorter than to BSA with dose-dependent manner (Fig. 2d & f). Double diffusion is reasonably easy to perform although its sensitivity is not very ideal. Whether appearance of “precipitation lines” or not could be dependent on the different conformations of complexes of thrombin/TBA and SA/Bio-ODN. Dissociation constant (Kd) for thrombin and TBA is 2–170 × 10^− 6^ M, but for SA and biotin is almost 10^− 15^ M [54, 55]. Detectable binding signal of protein target were observed ranging from 0.1 to 1.0 μg/μL by using the aptamer concentration of 10 μM in 5 μL volume system. With the increasing of length of the aptamer, the lower concentration such as 1 μM of 80 nt aptamer is also meet the demand for diffusion.

For small molecules, two binding systems (acetamiprid and profenofos) were selected for monitoring by double diffusion. Image analysis results showed that there was no significant difference between target-bound and target-free aptamer using double diffusion approach (Additional file 5: Figure S4). When the molecular weight of small molecule targets is far lower than that of their corresponding aptamer, they are not heavy enough to affect the diffusion of aptamer. It suggests that a novel approach is demanded for detection of aptamer-target binding when the target molecule weight is very low.

### Aptamer-based single diffusion

In single diffusion, the results showed that “precipitation ring” were easily observed in Bio-ODN and SA binding reaction (Additional file 6: Figure S5a). “Precipitation trace” rather than “precipitation ring” was found in thrombin-TBA binding groups (Additional file 6: Figure S5c) compared to the SA-Bio-ODN groups. This may be caused by the difference of affinity between TBA and Bio-ODN with theirs target molecules, which similar with double diffusion. Concentrations of protein target ranging from 0.5 to 1 μg/μL can cause the binding signals by using the aptamer concentration of 0.25 μM in gel. This kind of assay needs a larger volume system (almost 7 mL gels) with more aptamer to meet the requirement to form a visible diffusion difference. Though the sensitivity of this method is reasonable, not so suitable for its applications due to the large amount of aptamer required. Moreover, small molecules (i.e. acetamiprid) were used for testing the monitoring system, results showed that no significant difference was found (Additional file 7: Figure S6).

### Aptamer-based chasing diffusion

**Fig. 3:**
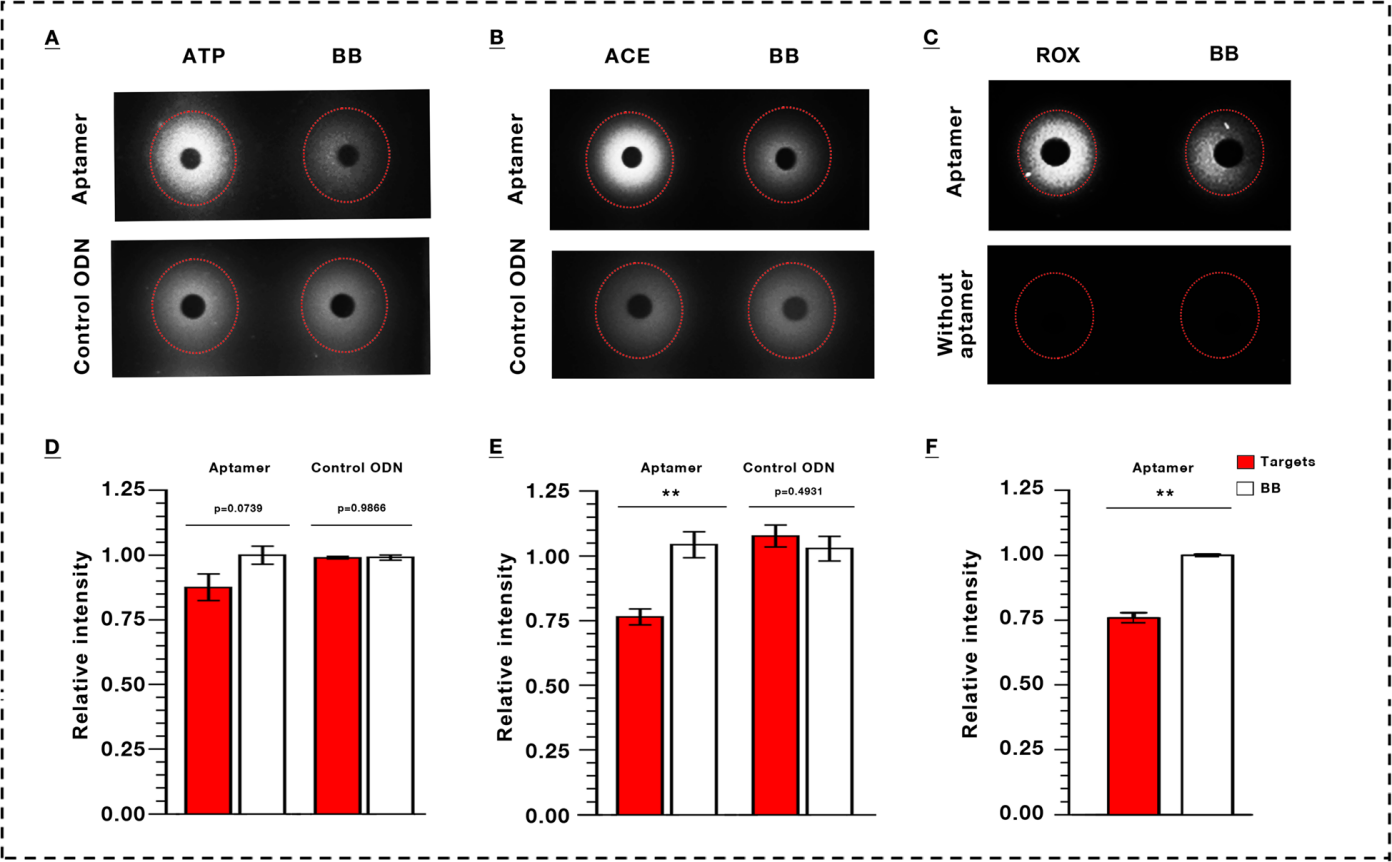
Characterization of aptamer-target binding by chasing diffusion in mini-gels. **a**, **b**, **c**, Binding signal of ATP, acetamiprid (ACE) and roxithromycin (ROX) with their aptamers or respective control oligodeoxynucleotides (ODN), respectively. **d**, **e**, **f**, Analysis of the intensity for **a**, **b**, **c**, respectively. Binding buffer (BB) as a negative control; Data represent mean ± SEM. **P < 0.01, set control = 1.0. SYBR Green I as the DNA indicator

The conformation of aptamer could be changed resulting from binding of aptamer and its target. Consequently, the number of complementally binding base pair may be changed, i.e. double-stranded regions will be different [50]. In chasing diffusion, the significant signal difference between the target molecules and control molecules were found in this study (Fig. 3, Additional file 8: Figure S7). In our previous works, we applied an ssDNA library immobilized SELEX protocol to isolation of aptamers binding to roxithromycin (ROX). One novel candidate aptamer was identified that binds to ROX with high affinity by using SYBR Green I assay (data unpublished). To validate our method, we firstly performed the chasing diffusion to monitoring the binding of aptamer with ROX. The significant signal difference between the ROX molecules and control molecules were found in this study (Fig. 3c & f).

The aptamer concentration of 10 μM in 5 μL volume system is enough for chasing diffusion. In principle, chasing diffusion used duplex-specific nucleic acid dye as indicator, which is same as the standard SGI assay [56]. Compared with standard SGI assay, the detection mode is changed from fluorescence to UV detection in our proposed gel-based method. This method does not require relatively sophisticated equipments and are user friendly with labor- and time- saving in experimental operation.

### Monitoring the enrichment of the dynamic libraries in SELEX rounds

Various approaches for monitoring the SELEX process have been reported [42, 57–75]. The most straightforward monitoring method involves the assessment of sequence enrichment in RNA pools, a technique made possible by the advent of HTS technology [42, 57–61]. However, HTS is expensive, and sample preparation for HTS is time-consuming. Another effective way of monitoring is to assess the average affinity of the RNA pools to the target molecule [42, 64–74]. Several such assessment methods are available, including SPR, EMSA, and the filter-binding assay. Furthermore, fluorescence-activated cell sorting (FACS) has recently been applied for monitoring the evolution of nucleic acid [73, 74].

**Fig. 4:**
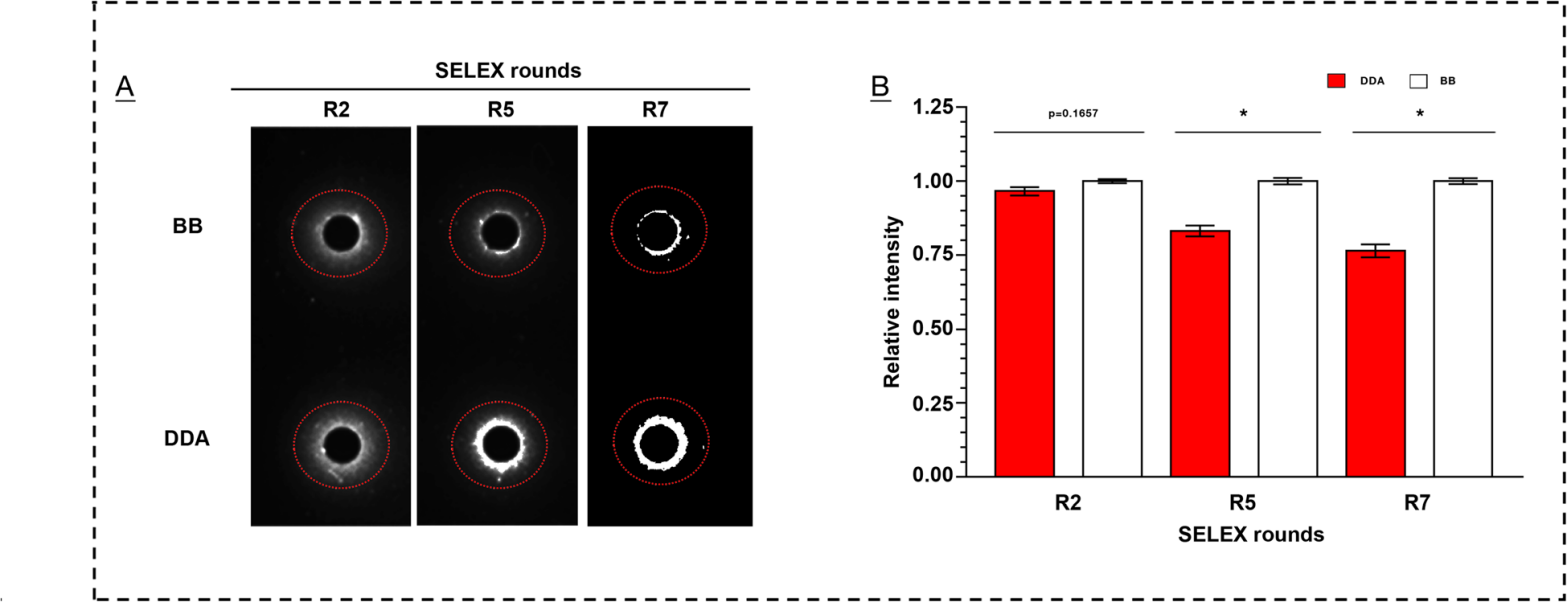
Monitoring the enriching situation of the dynamic libraries in SELEX process. **a** Binding signal of DDA with the libraries of the representative rounds; **b** analysis of the intensity. DDA: 2,2-bis(4-chlorophenyl) acetic acid; BB: binding buffer; Data represent mean ± SEM. *P < 0.05, set control =1.0. SYBR Green I as the DNA indicator

In the scenario that there is a significant difference of molecule weights between target (less than 500 Da) and aptamer, their diffusion rates must be significantly different. In many cases of double diffusion, the target molecules are too small to influence the diffusion distance of aptamer, even they bind each other with high affinity. Recently, we applied SELEX protocol to isolation of aptamers (almost 25.96 kD) bound to 2,2-bis(4-chlorophenyl) acetic acid (DDA) (281.134 Da). We firstly performed chasing diffusion to regularly monitor the enrichment of the library in SELEX process. In each round, the sample of library obtained from selection by candidate-target binding was loaded into the well first. After an optimized period, the target solution was then added into the well to chase the aptamer candidates. The significant signal difference between DDA molecules and control molecules were found since round 5. Three representative libraries were demonstrated in Fig. 4. It strongly implies the application potential of chasing diffusion. Real-time PCR also showed that the DDA specific elutions contain more DNAs than binding buffer washing controls since round 5 (Additional file 9: Figure S8b).

In selection of aptamers against DDA, the results are very encouraging [76]. One candidate aptamer bound to DDA with high affinity with low cross-binding activities on other small molecules were selected and detected by using SYBR Green I assay (Additional file 9: Figure S8c & d).

### A experimental summary of the reported methods used in SELEX rounds

A table including the average duration time, requires of normal reagents, cost and the sensitivity was summarized to better compare the methods used in SELEX (Table 2). GBDM is a low-cost and easy performed method with reasonable sensitivity. In additional, the method focuses on the binding phase of the aptamer candidates and the target, which shows the real enrichment state in the SELEX process. The diffusion of our method does take time, in practical, it can be done overnight for each round of selection without additional labor cost.

**Table 2:** Comparison of the reported methods used in SELEX rounds

Monitoring methods	Average duration time	Normal reagents required	Average cost in reagents per assay	Instrument and its average cost	Sensitivity	Focus on phase
UV quantification	0.5 h	~	~	UV quantification instrument 15,000 USD	ng	Eluting phase
GBDM	6 to 9 h	Nucleic acid dye	Below 1 USD	The user-defined mini gel cassette 15 USD UV imager 1500 USD	μg or nM	Binding phase
Gel-shifting	2 h	Nucleic acid dye	Below 1 USD	Electrophoresis equipment 1800 USD UV imager 1500 USD	μg	Binding phase
Agarose gel analysis	0.5 h	PCR reaction reagent; Nucleic acid dye	Below 1.5 USD	Electrophoresis equipment 1800 USD UV imager 1500 USD	ng	Eluting phase
qPCR	2 h	qPCR reaction reagent	25 USD	Real time PCR amplifier 30,000 USD	pg	Eluting phase
Fluorescence quantification	1 h	Fluorophore-labeled primer	25 USD	Fluorescence quantification instrument 70,000 to 150,000 USD	pg	Eluting phase
Dot blotting	7 h	Biotin labelled primer; NC membrane; SA-HRP reagents; ECL chemiluminescent solution	30 USD	Chemiluminescence apparatus 30,000 USD	ng	Binding phase
EMSA	7 h	Labeled aptamer (such asγ-32P and biotin); Nylon membrane; Detection reagents	30 USD	Electrophoresis equipment 1800 USD Imaging apparatus 50,000 USD	μg	Binding phase
ELONA	6 h	Biotin-labeled primer; ELISA plates; SA-HRP reagents; TMB solutions	30 USD	Microplate reader 20,000 USD	ng	Binding phase
SPR	6 h	Sensor chip	90 USD	SPR instrument 150,000 USD	pg	Binding phase
Gel-elution assay	7 h	Target-coupled column; PCR reaction reagent	N/A	Electrophoresis equipment 1800 USD UV imager 1500 USD	μg	Eluting phase

Notes: The methods ranked by average cost in reagents per assay; ~ means none cost; N/A means not applicable

*GBDM* gel-based diffusion method, *qPCR* real-time quantitative PCR, *EMSA* Electrophoretic mobility shift assay, *ELONA* enzyme-linked oligonucleotide assay, *SPR* Surface plasmon resonance

These results indicate that GBDM, in particular, chasing diffusion is suitable for monitoring the interaction between enriched aptamer candidates and their targets. This developed method is easy performed with reasonable sensitivity. Moreover, we invented a device using proposed mechanism for its better application.

## Materials & methods

All oligodeoxynucleotides (Additional file 10: Table S2) were synthesized and purified by High Performance Liquid Chromatography (HPLC, Sangon biotech, Shanghai, China). Thrombin (alpha) (Cat.No. HCT-0020, USA) was purchased from Haematologic Technologies Inc. (USA). Streptavidin (SA, Cat.No. S9170-1 mg), bovine serum albumin (BSA, Cat.No. A8020), goat anti-rabbit IgG (Cat.No. SPA134) and rabbit IgG (Cat.No. SP034) were purchased from Solarbio life sciences (Beijing, China). GelRed stain (Cat.No. KGM025R) was obtained from KeyGEN BioTECH (Nanjing, China). SYBR Green I (Cat.No. A502040–0500) was purchased from Sangon biotech (Shanghai, China). Acetamiprid (Cat.No. A109930-100 mg) was purchased from ALADDIN (Shanghai, China); Profenofos (Cat.No.1633000) was purchased from LGC Labor GmbH (Germany). Binding buffer A (20 mM Tris-HCL, pH 7.5, 100 mM NaCl, 2 mM MgCl_2_, 5 mM KCl, 1 mM CaCl_2_), binding buffer B (0.1 M PBS with 1 mM Mg^2+^), binding buffer C (300 mM NaCl, 5 mM MgCl_2_, 20 mM Tris, pH 7.6) and binding buffer D (10 mM NaCl, 10 mM KCl, 10 mM MgCl_2_ and 50 mM Tris/HCl, pH 8.0) was prepared for these assays. All other chemical reagents were analytically pure grades and purchased from Sinopharm Chemical Reagent Co., Ltd. (Shanghai, China). Water used in all experiments was Elix water.

### Preparation of mini gel cassette

The experimental models of gel cassette (Fig. 1) were designed by using Materialise 3-MATIC software (www.materialise.com) and fabricated by 3D printing technology (ZRapid Tech, iSLA550, China) using Stereo Lithography Apparatus (SLA) with transparent resin (ZRapid Tech, ZR580, China). Precision of printing was 0.1 mm.

In mini gel cassette, four parts could be assembled before making gel as the diagram in Fig. 1b. Briefly, the tray could be placed into the concave of the base, a smooth glass plate could be placed on the tray. Agarose with binding buffer were melted, mixed with GelRed or SYBR Green I stain (with aptamers for single diffusion) and then poured on the glass plate which was pre-assembled in the concave with tray. A suitable mold could be placed on the tray with melted agarose to make sample wells on the gel. After a half-hour setting, the mold was carefully removed off and the gel with sample wells was ready to be used for loading samples.

### Double immunodiffusion (DID) tests by using our mini gel cassette

Immunodiffusion tests were performed in 2.0% agarose. Each agar plate contained a five-well pattern. The wells were 0.85 mm in diameter. Each of the 4 wells in the outer side was spaced 4.5 mm from the centre well (Additional file 1: Figure S1). Reagents were added to the wells in a consistent pattern. 5 μL of each reference antiserum (goat anti-rabbit IgG) and of each reference antigen (Rabbit IgG) and the negative control were added to the designated well. Slides were incubated in a moist atmosphere at 37 °C. Readings were made after overnight incubation (16 h).

### GBDM for monitoring the binding of aptamer and target

A protocol for GBDM assay including a step-by-step guide to the procedure can be found in Additional file 11: Protocol.

#### Gel preparation

The 2.0–3.0% (m/v) agarose with binding buffer (pre-preparation refer to materials and methods, Additional file 10: Table S2) were melted, mixed with 1 × GelRed or 1× SYBR Green I and loaded into the device carefully. After a half-hour, gels are ready for monitoring after removing the hole-making molds carefully.

### Aptamer-based double diffusion

#### Principle of double diffusion

**Fig. 5:**
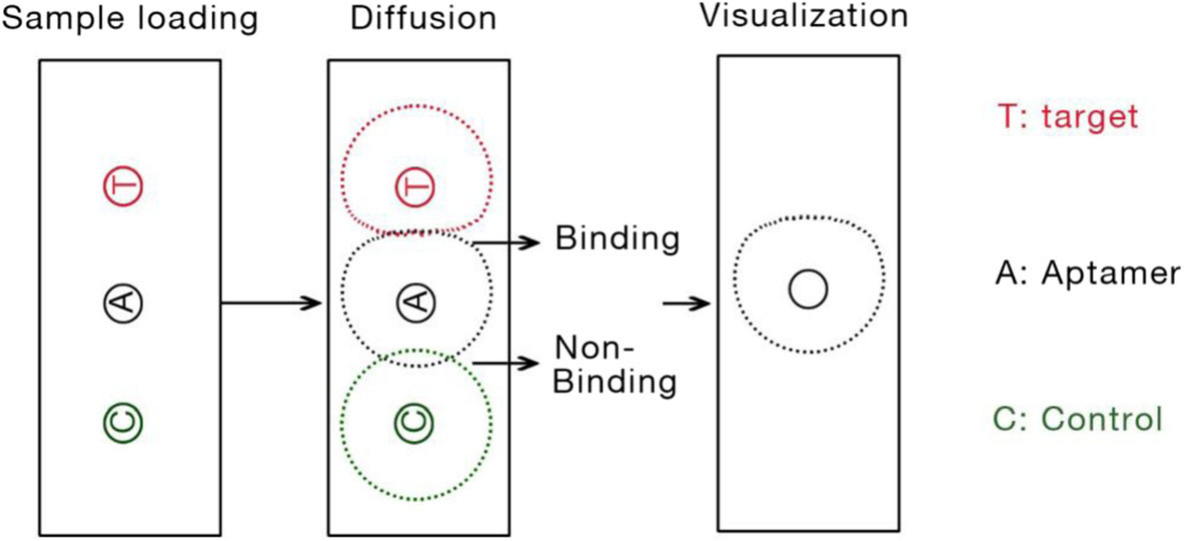
Schematic diagram of the aptamer-based double diffusion

There are three wells for each test. The middle one is for loading aptamer or control ODN sample. Both control materials and target molecules were simultaneously loaded in a designed well beside the aptamer/control ODN well in agarose gel respectively (Fig. 5). In the diffusion process, molecules including aptamer/control ODN, control material and target molecules are diffused freely in gel. Once target molecules meet with the aptamer, formation of aptamer-target complex reduces the diffusion speed. This difference can detect and visualize by UV-light system.

#### Double diffusion

Optimal diffusion conditions were determined at room temperature. Details in thrombin and SA assays: Concentration of TBA and Bio-ODN: 10 μM. Targets concentration: 1.0, 0.5, and 0.1 μg/μL. Diffusion time was 6 h at room temperature. Gel concentration: 2.0% in binding buffer. Control ODN and bovine serum albumin (BSA) as negative controls. Sample volume: 5 μL for each well.

### Aptamer-based single diffusion

#### Principle of single diffusion

**Fig. 6:**
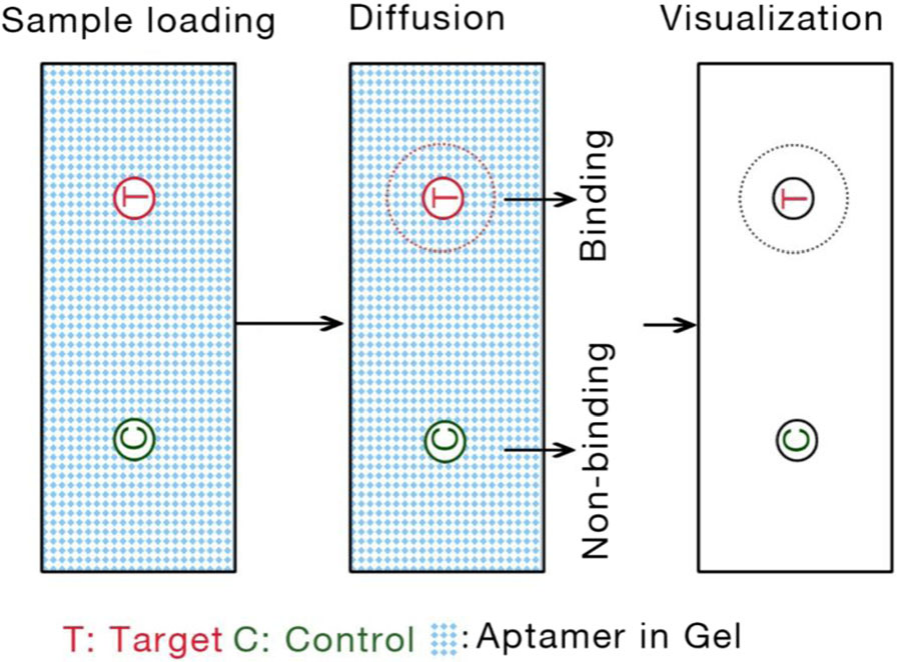
Schematic diagram of the aptamer-based single diffusion

For single diffusion, in gel preparation step, gels needed to be mixed with aptamer candidates or control ODN firstly. Then the target molecules and control materials were loaded for diffusion (Fig. 6). Once target molecules meet with the aptamer in gel, aptamer-target complex are constantly forming on the road. These aptamer-target complex can detect and visualize by UV-light system. Conversely, gels could be mixed with target molecules firstly.

#### Single diffusion

Optimal diffusion conditions were determined at room temperature. Gel concentration: 2.0% in binding buffer with aptamer candidates or control ODN. Details in thrombin and SA assays: Concentration of TBA and Bio-ODN: 0.25 μM. Targets concentration: 1.0, 0.5, and 0.1 μg/μL. Diffusion time was 16 h at room temperature. Control ODN and bovine serum albumin (BSA) as negative controls. Sample volume: 5 μL for each well.

### Aptamer-based chasing diffusion

#### Principle of chasing diffusion

**Fig. 7:**
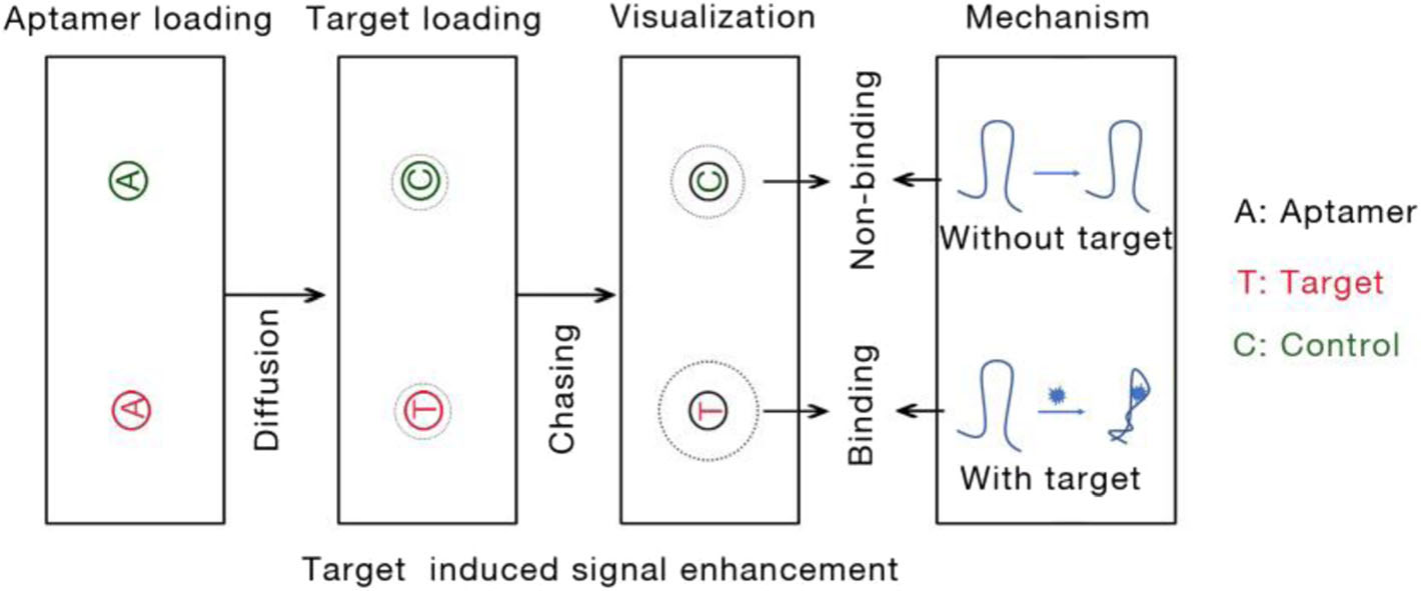
Schematic diagram of the aptamer-based chasing diffusion (signal enhancement)

Preparation of low molecular weight target molecule or control material was loaded into the well where the aptamer preparation was previously loaded and allowed to diffuse for a definite time (Fig. 7). Once target molecules meet with the aptamer in gel, formation of aptamer-target complex could induce the signal enhancement or weakness. This difference can detect and visualize by UV-light system.

#### Chasing diffusion

Optimal diffusion conditions for chasing diffusion were determined at room temperature. Concentration of Aptamer and Bio-ODN: 10 μM. Targets concentration: ATP, 10 mM; acetamiprid, 9.85 mM. Diffusion time was 9 h at room temperature including 30 min for aptamer diffusion. Control ODN or binding buffer only as a negative control. Gel concentration: 3.0% in binding buffer. Sample volume: 5 μL for each sample.

#### Image taken and analysis

Interaction of stained aptamer and target was visualized by photography of the gels with UV light using a gel imaging system (Tanon 4600SF, Shanghai, China). Images were processed and quantified by calculating distance or scanning densitometry using Image-pro plus (version 6.0) (www.mediacy.com) processing software and normalized to the signal intensity of control materials.

### Monitoring the enriching situation of library in SELEX process

Selection of an aptamer against DDA was performed by using a variation of the SELEX protocol (Additional file 9: Figure S8). In brief, the synthetic ssDNA library (80 nt with 40 nt random sequence) was dissolved in binding buffer. Biotin-labeled library complementary sequence was used to capture the library onto the streptavidin magnetic beads. The magnetic beads were washed 6 times, and incubated for 90 min with DDA in binding buffer (200 μL) to a final concentration of 100 μM at room temperature. The supernatant was collected by magnetic separation and monitored by real-time PCR. Then the DDA-specific elution was amplified by PCR and library was generated for next selection round. In the last selection round, the library was sequencing. Characterization of candidate aptamers for DDA was conducted by SYBR Green I assay.

In this study, the library obtained from each round was sampled and monitored by chasing diffusion. Concentration of sub-libraties: 700 nM to 1 μM. Gel concentration: 3.0% in binding buffer. Targets concentration: 10 μM. Diffusion time was 9 h at room temperature. Binding buffer only as a negative control. Sample volume: 5 μL. Library samples of round 2, 5 and 7 were duplicated and demonstrated in this report.

### Characterization of a novel aptamer against small molecule by using mini gel

To validate our concept of mini gel, a novel candidate aptamer against roxithromycin (ROX) was selected and monitored by mini gel. The candidate aptamer was newly selected and characterized by SYBR Green I assay with high affinity by our research group (data unpublished). Gel concentration: 3.0% in binding buffer. Candidate aptamer concentration: 5 μM; Targets concentration: 1 mM. Diffusion time was 9 h at room temperature. Binding buffer only as a negative control. Sample volume: 5 μL.

### Statistical analysis

All experiments in this study were performed at least in triplicate for each control and treatment group. The numeric data are expressed as the mean ± SEM. Differences between groups were evaluated using Student’s t test. *P* < 0.05 was considered statistically significant. GraphPad Prism 7 (version 7.00) (www.graphpad.com) was used to display and analyze the data.

## Conclusions

In summary, the GBDM described here is a kind of convenient aptamer-target binding assay. Both double diffusion and single diffusion are good to monitor the binding behavior of macromolecule target. Whereas, the developed chasing diffusion is recommended for monitoring the interaction between dynamic aptamer libraries and their targets, in particular for small molecule targets, round by round during the process of SELEX. Our study and theory will yield benefits in discovery of novel aptamers, the modern non-animal affinity recognition elements of aptasensors.

## Supplementary information

**Additional file 1:** **Figure S1.** Excellent performance in double immunodiffusion (DID) experiment by using designed gel cassette. Stars: Precipitation line; Ag, rabbit IgG (10 mg/mL); Ab, goat anti-rabbit IgG (different ratio in PBS buffer); C, PBS as negative control; Diffusion time was 16 h at 37 °C.

**Additional file 2:** **Table S1.** Optimizations of the diameter of different well sizes and their maximum volumes for sample loading.

**Additional file 3:** **Figure S2.** Optimization (A) and analysis (B) of the diffusion distance by using streptavidin (SA) and biotinylated oligodeoxynucleotides (Bio-ODN) by double diffusion. Arrows: target-DNA binding complexes. Concentration of Bio-ODN: 5 μM. Targets concentration: 1.0 μg/μL. Diffusion time was 6 h at room temperature. Bovine serum albumin (BSA) as a negative control.

**Additional file 4:** **Figure S3.** There is no significant difference in diffusion situation of oligodeoxynucleotides (ODN) in non-horizontal placed gel. The gel was placed in erect direction after 5 min incubation for diffusion. It has little impact in the diffusion pattern of the ODN. Diffusion time was 6 h at room temperature.

**Additional file 5:** **Figure S4.** There are no binding signals in aptamer- profenofos (A) and -acetamiprid (B) by double diffusion, respectively. Concentration of Aptamers: 10 μM. Targets concentration: profenofos,10 mM; acetamiprid, 9.85 mM. Diffusion time was 6 h at room temperature. Binding buffer as a negative control.

**Additional file 6:** **Figure S5.** Characterization of aptamer-target binding by single diffusion in mini-gels. A and B, Binding signal of streptavidin (SA) and biotinylated oligodeoxynucleotides (Bio-ODN) (A) and control DNA (B). C, Analysis of diffusion radius centered at the centre of the well for SA. D and E, Binding signal of thrombin and its aptamer (TBA) (D) and control DNA (E). F, Analysis of diffusion radius centered at the centre of the well for thrombin. Arrow heads in A: diffusion ring; Thin arrow heads in D: diffusion trace. Data represent mean ± SEM. *P < 0.05, **P < 0.01, set control = 1.0. GelRed as the DNA indicator.

**Additional file 7:** **Figure S6.** There is no binding signal in aptamer-acetamiprid by single diffusion. Concentration of Apt: 0.25 μM. Targets concentration: 8.95 mM. Diffusion time was 16 h at room temperature. Binding buffer as a negative control. The abnormal high intensity spot at the top left well was caused by a bubble.

**Additional file 8:** **Figure S7.** Characterization of the binding of aptamer-profenofos and -atrazine by chasing diffusion. Concentration of Apt: 10 μM. Targets concentration: profenfos, 10 mM; atrazine, 20 mM; Diffusion time was 9 h at room temperature. Binding buffer only as a negative control.

**Additional file 9:** **Figure S8.** Generation and characterization of aptamers against DDA. A, SELEX protocol to isolation of aptamers bound to DDA; B, Enrichment of the dynamic libraries in SELEX rounds by real-time PCR; C and D, characterization of the affinity (C) and selectivity (D) of one aptamer against DDA by using SYBR Green I assay. BB:Binding buffer; DDA: 2,2-bis(4-chlorophenyl) Acetic Acid; DDT: 2,4′-DDT; PFOA: Perfluorooctanoic Acid; EP: Ethyl pyruvate; TBBPA: Tetrabromobisphenol A; Data represent mean ± SEM. *P < 0.05, **P < 0.01, ***P < 0.001.

**Additional file 10:** **Table S2.** Sequences of oligodeoxynucleotides (ODN) used in this study.

**Additional file 11: PROTOCOL.** A protocol for GBDM assay including a step-by-step guidance.

## Data Availability

All data generated or analyzed in this study are included in this published article, its supplementary information files, and additional files.

## Abbreviations

Bio-ODN: Biotinylated oligodeoxynucleotides
CE: Capillary electrophoresis
DDA: 2,2-bis(4-chlorophenyl) acetic acid
DID: Double immunodiffusion
ELONA: Enzyme-linked oligonucleotide assay
EMSA: Electrophoretic mobility shift assay
FACS: Fluorescence-activated cell sorting
GBDM: Gel-based diffusion method
HTS: High throughput sequencing
ITC: Isothermal titration calorimetry
ODN: Oligodeoxynucleotides
QCM: Quartz crystal microbalance
ROX: Roxithromycin
SA: Streptavidin
SELEX: Systematic Evolution of Ligands by EXponential enrichment
SLA: Stereo Lithography Apparatus
SPR: Surface plasmon resonance
TBA: Thrombin binding aptamer
