# Supplementary figures and images for: SELEX tool: a novel and convenient gel-based diffusion method for monitoring of aptamer-target binding
Source: J Biol Eng. 2020 Jan 13;14:1. doi: 10.1186/s13036-019-0223-y (PMC6956507; doi:10.1186/s13036-019-0223-y)

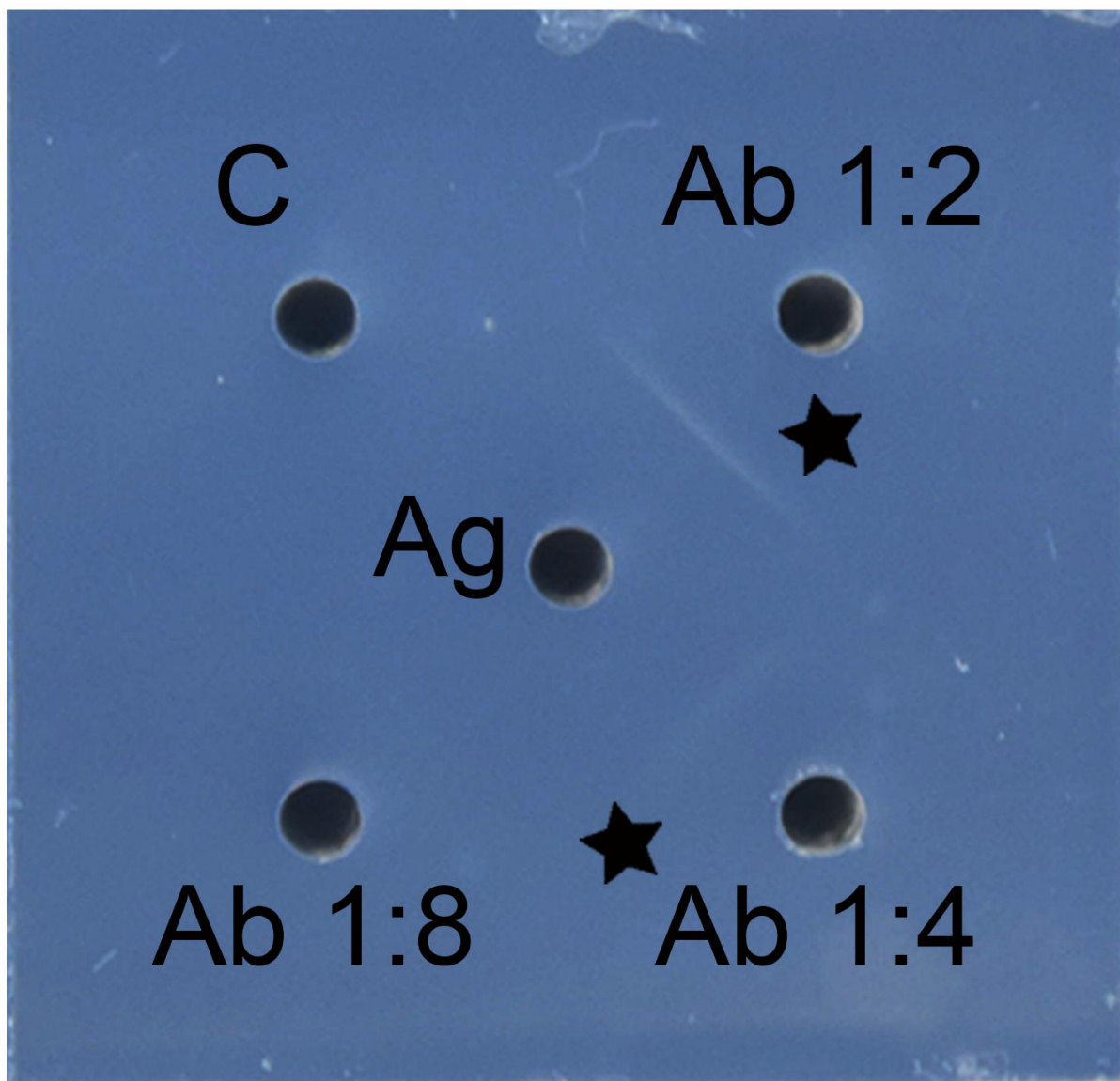

**Additional file 1 Figure S1**

Supplement: Supplementary file 1 — Additional file 1: Figure S1. Excellent performance in double immunodiffusion (DID) experiment by using designed gel cassette. Stars: Precipitation line; Ag, rabbit IgG (10 mg/mL); Ab, goat anti-rabbit IgG (different ratio in PBS buffer); C, PBS as negative control; Diffusion time was 16 h at 37 °C. [file 13036_2019_223_MOESM1_ESM.pdf]

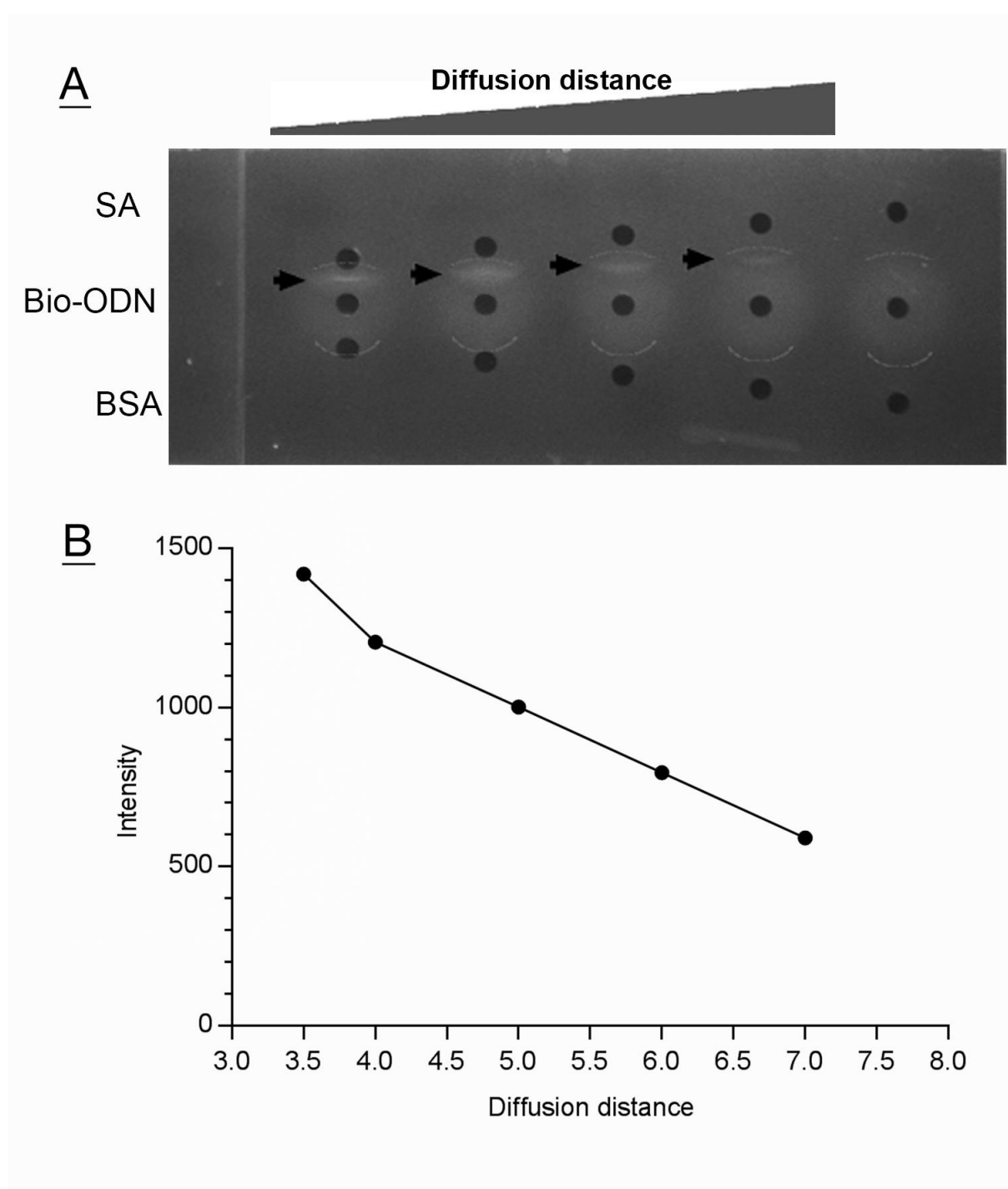

**Additional file 3 Figure S2**

Supplement: Supplementary file 3 — Additional file 3: Figure S2. Optimization (A) and analysis (B) of the diffusion distance by using streptavidin (SA) and biotinylated oligodeoxynucleotides (Bio-ODN) by double diffusion. Arrows: target-DNA binding complexes. Concentration of Bio-ODN: 5 μM. Targets concentration: 1.0 μg/μL. Diffusion time was 6 h at room temperature. Bovine serum albumin (BSA) as a negative control. [file 13036_2019_223_MOESM3_ESM.pdf]

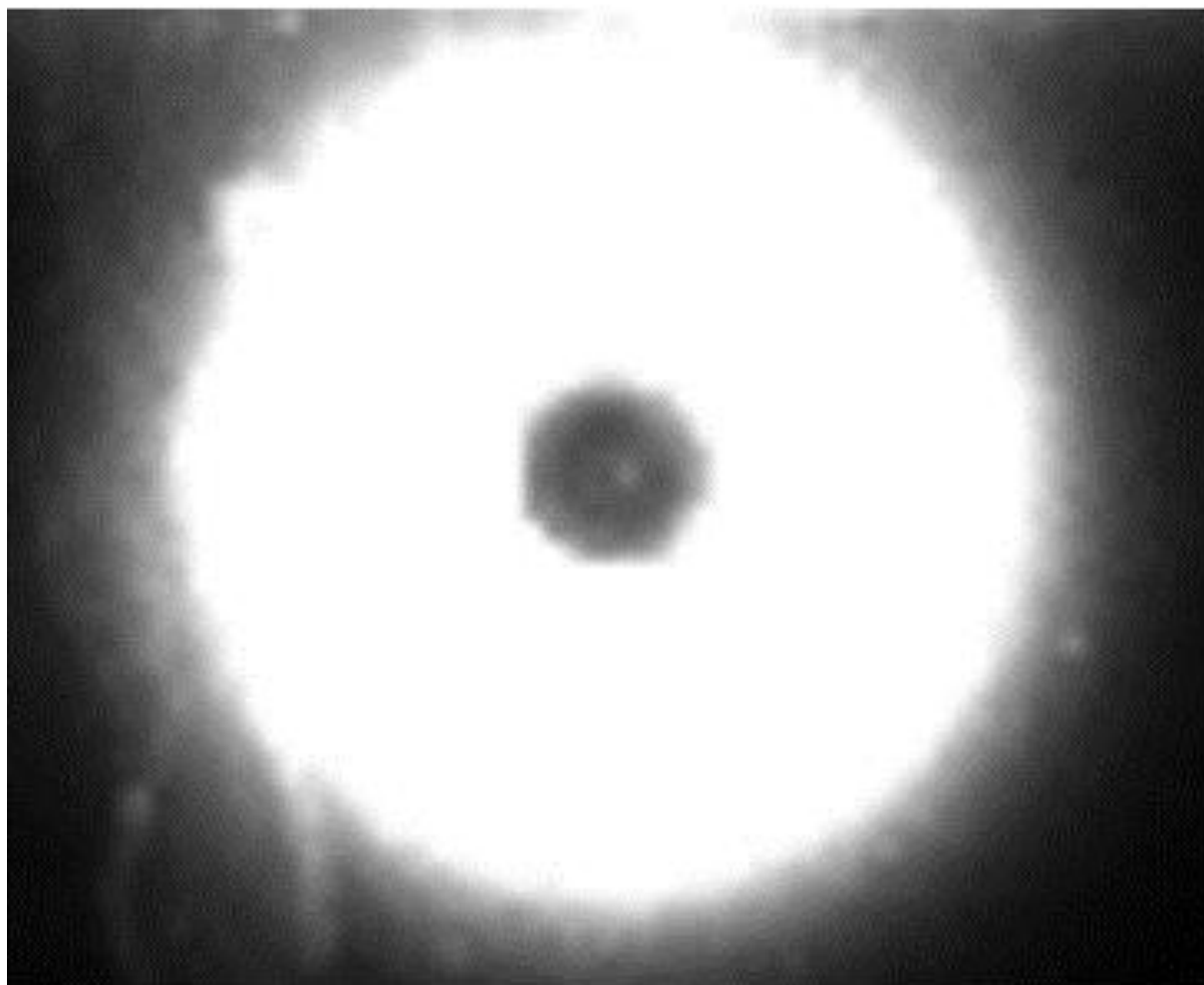

**Additional file 4 Figure S3**

Supplement: Supplementary file 4 — Additional file 4: Figure S3. There is no significant difference in diffusion situation of oligodeoxynucleotides (ODN) in non-horizontal placed gel. The gel was placed in erect direction after 5 min incubation for diffusion. It has little impact in the diffusion pattern of the ODN. Diffusion time was 6 h at room temperature. [file 13036_2019_223_MOESM4_ESM.pdf]

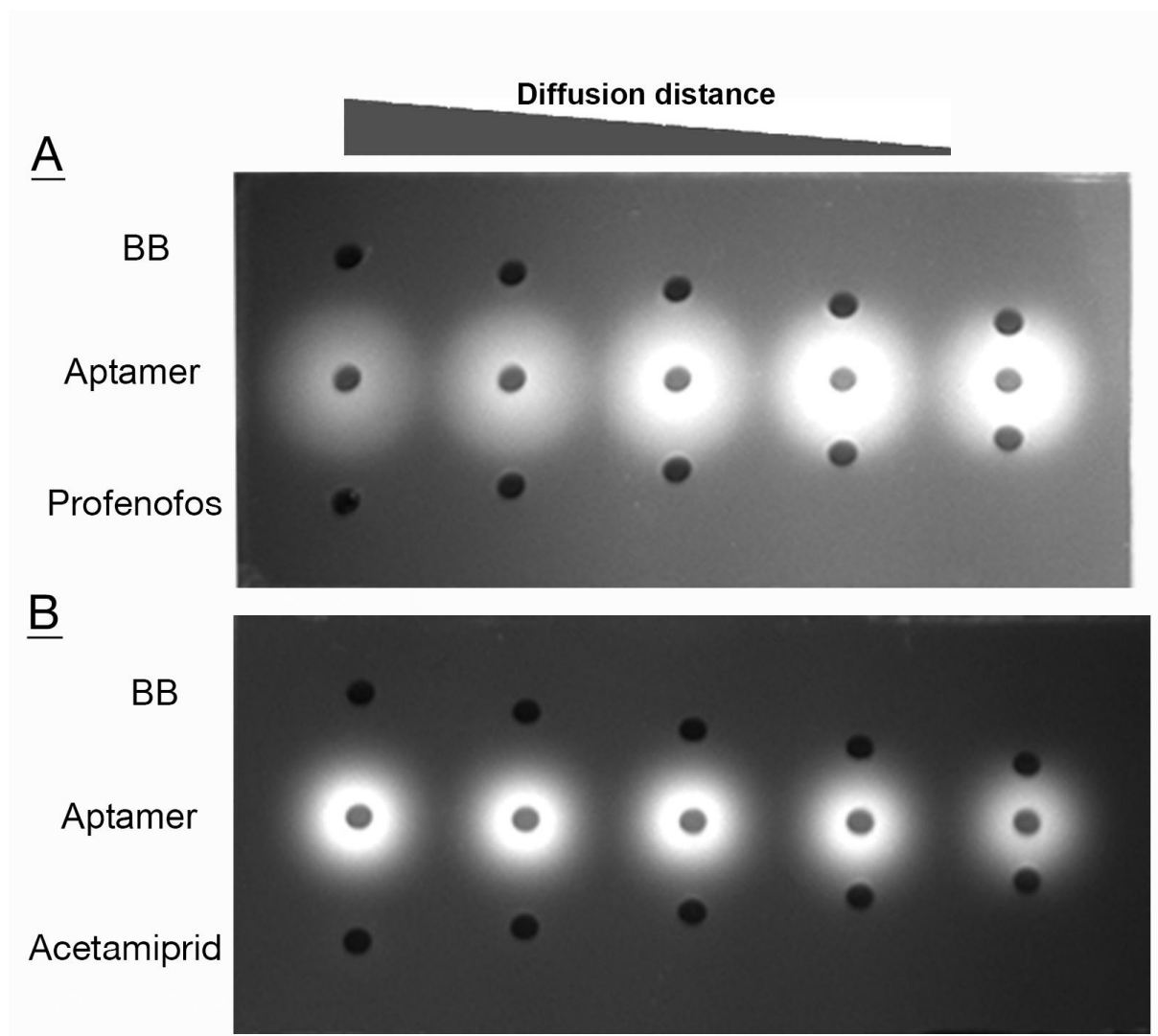

**Additional file 5 Figure S4**

Supplement: Supplementary file 5 — Additional file 5: Figure S4. There are no binding signals in aptamer- profenofos (A) and -acetamiprid (B) by double diffusion, respectively. Concentration of Aptamers: 10 μM. Targets concentration: profenofos,10 mM; acetamiprid, 9.85 mM. Diffusion time was 6 h at room temperature. Binding buffer as a negative control. [file 13036_2019_223_MOESM5_ESM.pdf]

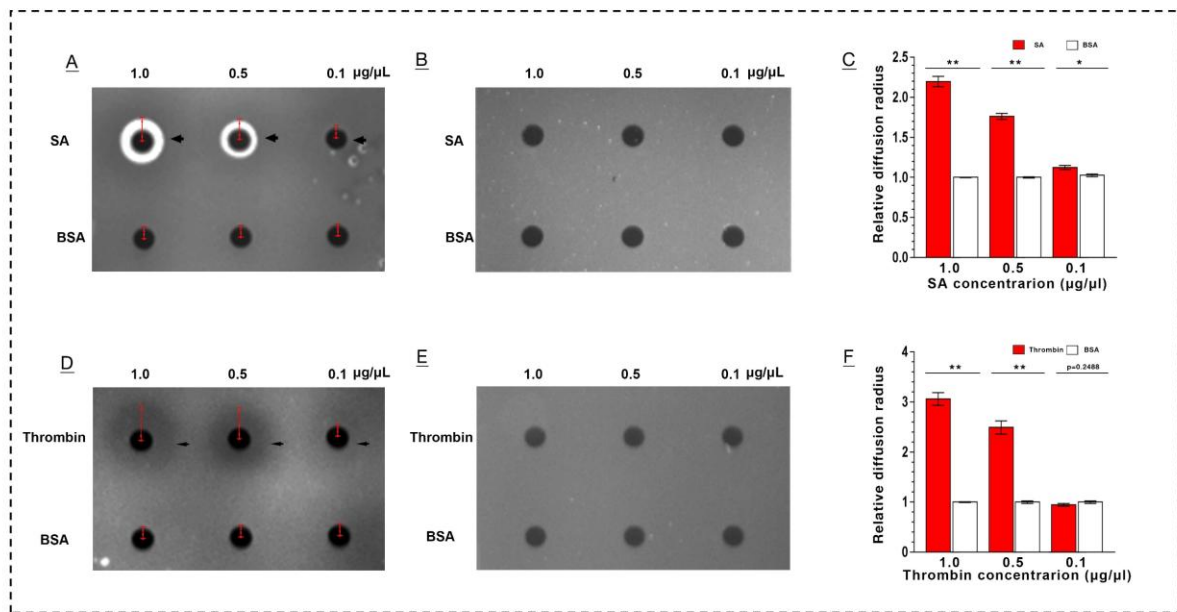

**Additional file 6 Figure S5**

Supplement: Supplementary file 6 — Additional file 6: Figure S5. Characterization of aptamer-target binding by single diffusion in mini-gels. A and B, Binding signal of streptavidin (SA) and biotinylated oligodeoxynucleotides (Bio-ODN) (A) and control DNA (B). C, Analysis of diffusion radius centered at the centre of the well for SA. D and E, Binding signal of thrombin and its aptamer (TBA) (D) and control DNA (E). F, Analysis of diffusion radius centered at the centre of the well for thrombin. Arrow heads in A: diffusion ring; Thin arrow heads in D: diffusion trace. Data represent mean ± SEM. *P < 0.05, **P < 0.01, set control = 1.0. GelRed as the DNA indicator. [file 13036_2019_223_MOESM6_ESM.pdf]

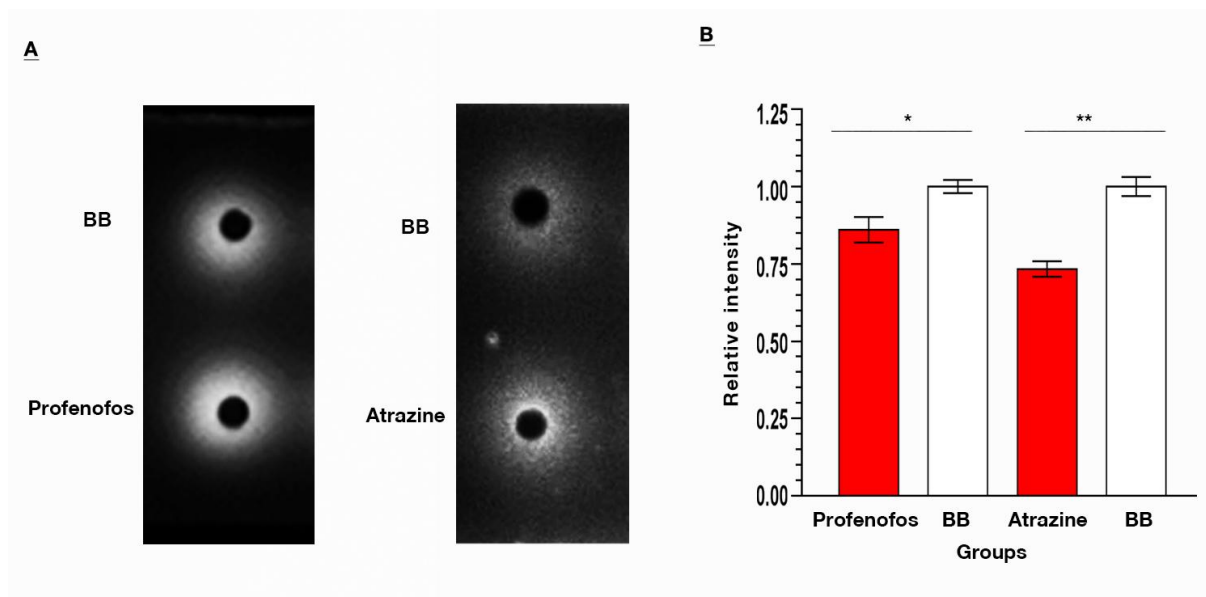

**Additional file 8 Figure S7**

Supplement: Supplementary file 8 — Additional file 8: Figure S7. Characterization of the binding of aptamer-profenofos and -atrazine by chasing diffusion. Concentration of Apt: 10 μM. Targets concentration: profenfos, 10 mM; atrazine, 20 mM; Diffusion time was 9 h at room temperature. Binding buffer only as a negative control. [file 13036_2019_223_MOESM8_ESM.pdf]

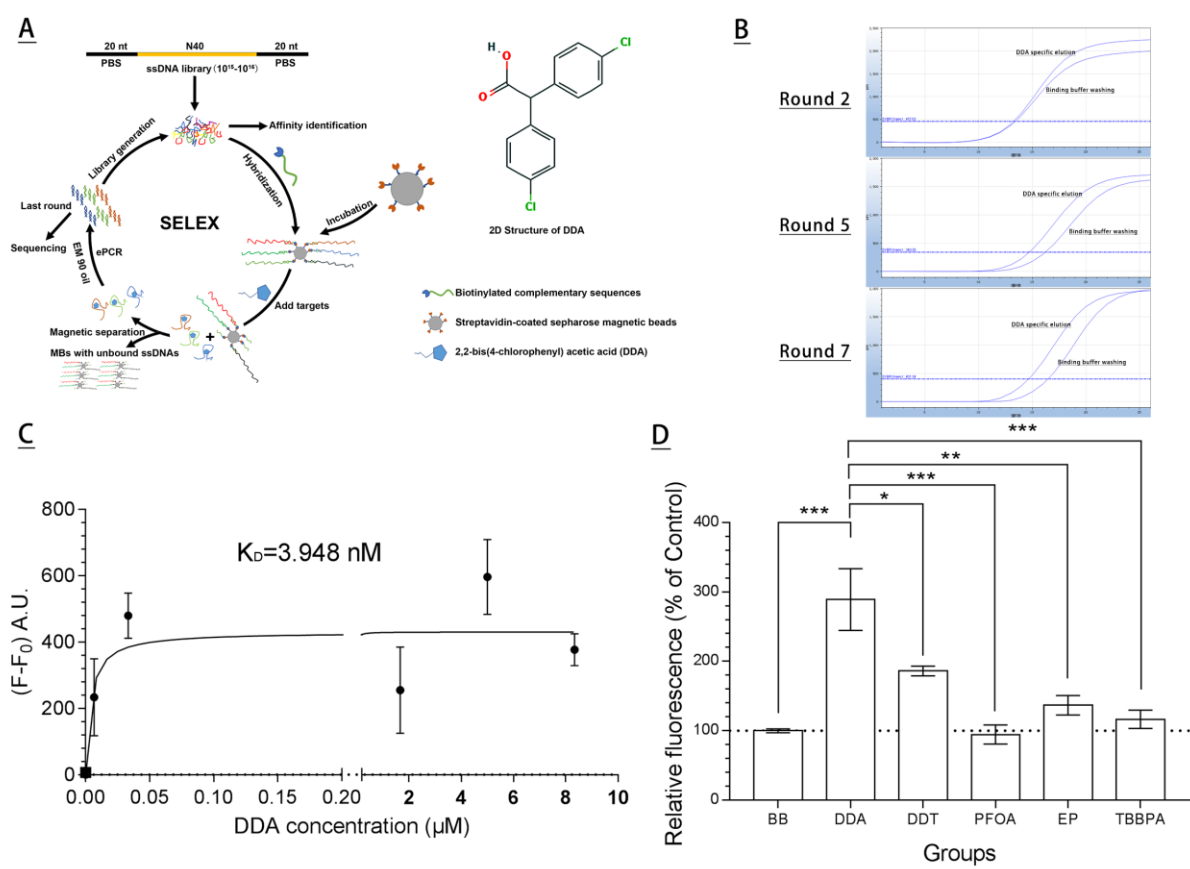

Additional file 9 Figure S8

Supplement: Supplementary file 9 — Additional file 9: Figure S8. Generation and characterization of aptamers against DDA. A, SELEX protocol to isolation of aptamers bound to DDA; B, Enrichment of the dynamic libraries in SELEX rounds by real-time PCR; C and D, characterization of the affinity (C) and selectivity (D) of one aptamer against DDA by using SYBR Green I assay. BB:Binding buffer; DDA: 2,2-bis(4-chlorophenyl) Acetic Acid; DDT: 2,4′-DDT; PFOA: Perfluorooctanoic Acid; EP: Ethyl pyruvate; TBBPA: Tetrabromobisphenol A; Data represent mean ± SEM. *P < 0.05, **P < 0.01, ***P < 0.001. [file 13036_2019_223_MOESM9_ESM.pdf]
