# Supplementary material for: SELEX tool: a novel and convenient gel-based diffusion method for monitoring of aptamer-target binding
Source: J Biol Eng. 2020 Jan 13;14:1. doi: 10.1186/s13036-019-0223-y (PMC6956507; doi:10.1186/s13036-019-0223-y)
Supplement: Supplementary file 2 — Additional file 2: Table S1. Optimizations of the diameter of different well sizes and their maximum volumes for sample loading. [file 13036_2019_223_MOESM2_ESM.doc]

# Table S1. Optimizations of the diameter of different well sizes and their maximum volumes for sample loading.

| Items | 1 | 2 | 3 | 4 |
| --- | --- | --- | --- | --- |
| Diameter (mm) | 0.5 | 0.75 | 0.85 | 1.00 |
| Maximum volume(μL) | 3.0 | 4.5 | 5.0 | 6.0 |

Diameter of 0.5 and 0.75 mm are too small for sample loading by using 10 μl tips.
