# Supplementary material for: SELEX tool: a novel and convenient gel-based diffusion method for monitoring of aptamer-target binding
Source: J Biol Eng. 2020 Jan 13;14:1. doi: 10.1186/s13036-019-0223-y (PMC6956507; doi:10.1186/s13036-019-0223-y)
Supplement: Supplementary file 7 — Additional file 7: Figure S6. There is no binding signal in aptamer-acetamiprid by single diffusion. Concentration of Apt: 0.25 μM. Targets concentration: 8.95 mM. Diffusion time was 16 h at room temperature. Binding buffer as a negative control. The abnormal high intensity spot at the top left well was caused by a bubble. [file 13036_2019_223_MOESM7_ESM.pdf]

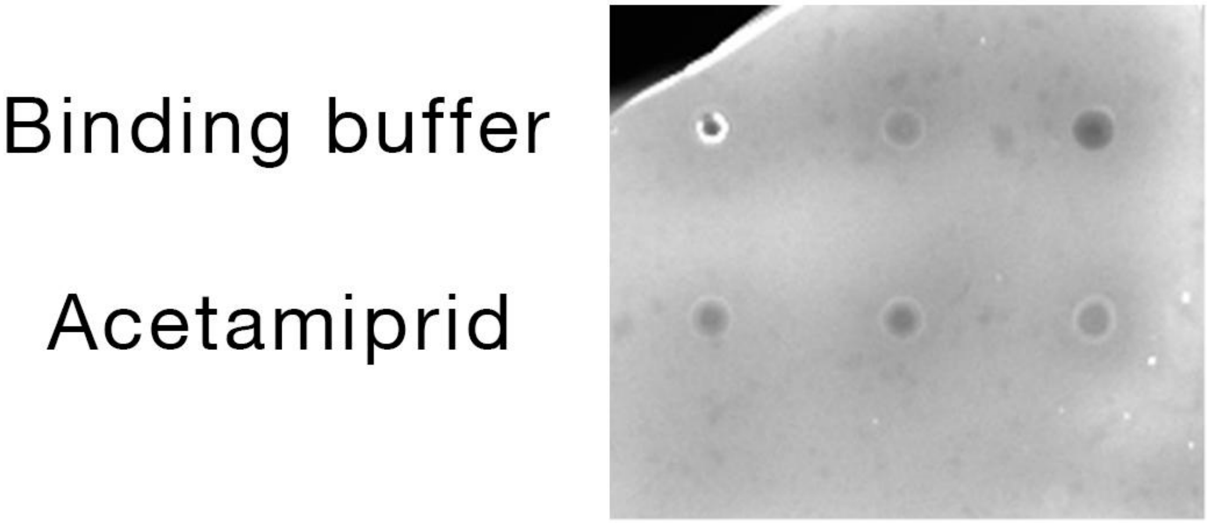

Additional file 7 Figure S6

Note: The abnormal high intensity spot at the top left well was caused by a bubble.
