# Supplementary material for: SELEX tool: a novel and convenient gel-based diffusion method for monitoring of aptamer-target binding
Source: J Biol Eng. 2020 Jan 13;14:1. doi: 10.1186/s13036-019-0223-y (PMC6956507; doi:10.1186/s13036-019-0223-y)
Supplement: Supplementary file 10 — Additional file 10: Table S2. Sequences of oligodeoxynucleotides (ODN) used in this study. [file 13036_2019_223_MOESM10_ESM.doc]

**Table S3. Sequences of oligodeoxynucleotides (ODN) used in this study.**

| Sample | Sequence (5’-3’) | Length of base pair | Modification | Reference | Binding buffer type |
| --- | --- | --- | --- | --- | --- |
| TBA | TTTTTTTTTTTTTTTGGTTGGTGTGGTTGG | 30 |  | [1] | A |
| TBA_Control DNA | CCAACCACACCAACCAAAAAAAAAAAAAAA | 30 |  |  | A |
| Bio-TBA | TTTTTTTTTTTTTTTGGTTGGTGTGGTTGG | 30 | 5’, Biotin |  | A |
| Bio-Oligo | AGCTTGCTGCAGCGATTCTTGATCGCCACAGAGCT | 35 | 5’, Biotin |  | A |
| Oligo_Control DNA | AGCTTGCTGCAGCGATTCTTGATCGCCACAGAGCT | 35 |  |  | A |
| Apt_acetamiprid | TGTAATTTGTCTGCAGCGGTTCTTGATCGCTGACACCATATTATGAAGA | 49 |  | [2] | B |
| Acetamiprid_Control DNA |  | 49 |  |  | B |
| Apt_atrazine | TCATGTTTGCACTGGCGGATTTAGCCAGTCAGT | 33 |  | [3] | B |
| Apt-ATP | CGCACCTGGGGGAGTATTGCGGAGGAAGGTGCG | 33 |  | [4] | C |
| ATP_Control DNA | CGCACCTTCCTCCGCAATACTCCCCCAGGTGCG | 33 |  |  | C |
| Apt_profenfos | AGCTTGCTGCAGCGATTCTTGATCGCCACAGAGCT | 35 |  | [5] | D |

All oligonucleotides were synthesized and purified by HPLC. Binding buffer types refer to REAGENTS AND MATERIALS.

1. Trapaidze A BA, Brut M.: **Binding modes of thrombin binding aptamers investigated by simulations and experiments**. *Applied Physics Letters* 2015, **106**(4):043702.

2. Fei AR, Liu Q, Huan J, Qian J, Dong XY, Qiu BJ, Mao HP, Wang K: **Label-free impedimetric aptasensor for detection of femtomole level acetamiprid using gold nanoparticles decorated multiwalled carbon nanotube-reduced graphene oxide nanoribbon composites**. *Biosens Bioelectron* 2015, **70**:122-129.

3. Madianos L, Tsekenis G, Skotadis E, Patsiouras L, Tsoukalas D: **A highly sensitive impedimetric aptasensor for the selective detection of acetamiprid and atrazine based on microwires formed by platinum nanoparticles**. *Biosens Bioelectron* 2018, **101**:268-274.

4. Huizenga DE, Szostak JW: **A DNA aptamer that binds adenosine and ATP**. *Biochemistry* 1995, **34**(2):656-665.

5. Zhang CZ, Wang L, Tu Z, Sun X, He QH, Lei ZJ, Xu CX, Liu Y, Zhang X, Yang JY *et al*: **Organophosphorus pesticides detection using broad-specific single-stranded DNA based fluorescence polarization aptamer assay**. *Biosens Bioelectron* 2014, **55**:216-219.
