# Supplementary material for: SELEX tool: a novel and convenient gel-based diffusion method for monitoring of aptamer-target binding
Source: J Biol Eng. 2020 Jan 13;14:1. doi: 10.1186/s13036-019-0223-y (PMC6956507; doi:10.1186/s13036-019-0223-y)
Supplement: Supplementary file 11 — Additional file 11: PROTOCOL. A protocol for GBDM assay including a step-by-step guidance. [file 13036_2019_223_MOESM11_ESM.doc]

# PROTOCOL

Include a step-by-step guide to the procedure.

1. Assembly of the device

Place the four parts including one base, one tray, a smooth glass slide, a number of hole-making molds according the guidance in Figure 2B.

**Attention**: Our experience is that preparing bubbles-free gels, hole-making molds should put in the middle of the base.

2. Gels preparation

For double diffusion and chasing diffusion, the 2% to 3% (m/v) agarose with binding buffer (pre-preparation see reagents and materials) were melted, mixed with 1×GelRed or 1× SYBR Green I and loaded into the device carefully. For single diffusion in gel, gels needed mixed with aptamers firstly.

This method is described for our optimized data. Gels concentration ranging from 1% to 3% are suitable for monitoring. Users will need to modify the binding buffer (according to the aptamer and target), the nucleic acid dye and the gel concentration as needed for monitoring.

**Attention**: At this stage, to avoid excessive volatilization, agarose should melt by intermittent heating such as 10s cycles. In addition, when adding the aptamers for Single diffusion, the temperature should keep at 65 ℃.

3. Remove the hold-making molds

After a half-hour, gels are ready for monitoring after removing the hole-making molds carefully.

4. Pretreatment of the aptamer

For double diffusion and chasing diffusion, aptamers (10 μM) in binding buffer were heated at 95 ℃ for 10 min, and then immediately put them in ice-water bath for 10 min.

**Attention**: At this step, aptamers can also heat at 70 ℃ or other temperature.

5. Sample loading

5 μL of both aptamer and target molecules were loaded in a well in agarose gel respectively. For single diffusion, target molecules were loaded in a well for monitoring.

**Attention**: When loading the samples, we need to replace the white tips each time to prevent the samples from spilling out of the holes, especially for air bubbles easily-formed samples such as BSA samples.

6. Diffusion

The diffusion was conducted in a humidifying box and the diffusion duration was 3-16 hours.

Diffusion duration described here were our optimized data. Diffusion duration ranging from 3 h to 6 h are suitable for double diffusion, 16 h for single diffusion, 9 h for chasing diffusion, but 24 hours the nucleic acid staining will disappear basically. For Users will need to modify it according to the properties for aptamers and targets.

7. Image taken and analysis

Then the gels were taken out carefully and the images were taken using a gel imaging system (Tanon 4600SF, Shanghai, China).

**Attention**: Bottom of gels are air bubbles free and the gels needed at less 1-5 min for restoring the shape after placing them on the platform for imaging.
